# Carbohydrate-Active Enzymes: Structure, Activity, and Reaction Products

**DOI:** 10.3390/ijms21082727

**Published:** 2020-04-15

**Authors:** Stefano Benini

**Affiliations:** Bioorganic Chemistry and Bio-Crystallography laboratory (B2Cl), Libera Università di Bolzano, 39100 Bolzano, Italy; Stefano.Benini@unibz.it

Carbohydrate-active enzymes are responsible for both the biosynthesis and breakdown of carbohydrates and glycoconjugates. They are involved in many metabolic pathways, in the biosynthesis and degradation of various biomolecules, such as bacterial exopolysaccharides, starch, cellulose, and lignin, and in the glycosylation of proteins and lipids. Their interesting reactions have attracted the attention of researchers belonging to different scientific fields ranging from basic research to biotechnology. Interest in carbohydrate-active enzymes is due not only to their ability to build and degrade biopolymers, which is highly relevant in biotechnology, but also because they are involved in bacterial biofilm formation, and in the glycosylation of proteins and lipids, which has important health implications. 

This Special Issue features a collection of research papers and reviews representing an up-to-date state of the art in this growing field of research to broaden our understanding about carbohydrate-active enzymes, their mutants, and their reaction products at the molecular level. 

Tindara Venuto et al. wrote a paper about alpha2,8-sialyltransferases from ray-finned fish and showed that, in tetrapods, duplicated *st8sia* genes like *st8sia7*, *st8sia8*, and *st8sia9* have disappeared while orthologues are maintained in teleosts [1]. They reconstructed the evolutionary history of *st8sia* genes in fish genomes and by bioinformatics analysis showed changes in the conserved polysialyltransferase domain of the fish sequences possibly accounting for variable enzymatic activities [1]. 

PhNah20A, a β-N-acetylhexosaminidase from the marine bacterium *Paraglaciecola hydrolytica* S66T, was identified and characterized. By phylogenetic analysis, the authors discovered that PhNah20A is located outside clusters of other studied β-N-acetylhexosaminidases, in a unique position between bacterial and eukaryotic enzymes. PhNah20A is the first characterized member of a distinct subgroup within GH20 β-N-acetylhexosaminidases. The recombinant PhNah20A showed optimum hydrolytic activity on GlcNAc β-1,4 and β-1,3 linkages in chitobiose (GlcNAc)2 and GlcNAc-1,3-β-Gal-1,4-β-Glc (LNT2), the core structure of a human milk oligosaccharide, at pH 6.0 and 50 °C. Interestingly PhNah20A catalyzed the formation of LNT2, the non-reducing trisaccharide β-Gal-1,4-β-Glc-1,1-β-GlcNAc, and in low amounts the β-1,2- or β-1,3-linked trisaccharide β-Gal-1,4(β-GlcNAc)-1,x-Glc by a transglycosylation of lactose using 2-methyl-(1,2-dideoxy-α-d-glucopyrano)-oxazoline (NAG-oxazoline) as the donor [2]. 

The production of biofuels from lignocellulosic biomasses is hampered by the saccharification step of the biomasses requiring the concerted action of lignocellulose cellulases and hemicellulases.

Zhang et al. characterized the secretome of *Trichoderma harzianum* EM0925 under induction of lignocellulose cellulases and hemicellulases [3]. Compared with the commercial enzyme preparations, the *T. harzianum* EM0925 enzyme cocktail presented significantly higher lignocellulolytic enzyme activities and hydrolysis efficiency against lignocellulosic biomasses. The enzyme mixture used on ultrafine grinding and alkali pretreated corn stover yielded 100% glucose and xylose. The authors suggest that natural cellulases mixed with the hemicellulases enzyme cocktail from *T. harzianum* EM0925 used for complete conversion of lignocellulose biomasses have a great potential for industrial applications [3]. 

BaAG2 from the early-diverged yeast *Blastobotrys (Arxula) adeninivorans* is an α-glucosidase of the GH13 family. The enzyme was overexpressed in *Escherichia coli*, purified, and characterized. BaAG2’s preferred substrates were maltose, other maltose-like molecules and malto-oligosaccharides with degrees of polymerization (DP) up to 4‒7, and sucrose, whereas isomaltose and isomaltose-like substrates were not hydrolyzed, confirming that BaAG2 is a maltase. At high maltose concentrations, BaAG2 exhibited transglycosylating ability, producing di- and trisaccharides. BaAG2 was inhibited by acarbose, Tris, and also by isomaltose-like sugars and glucose [4]. 

Levansucrases (LSCs, EC 2.4.1.10) are enzymes studied for their potential use in biotechnological application for the production of fructooligosaccharides and the transfructosylation of a wide range of acceptors. While LSCs from Gram-positive bacteria can produce long-chain levan (e.g., *Bacillus megaterium* SacB), LSCs from Gram-negative bacteria such as *Erwinia tasmaniensis* (EtLSC) are interesting biocatalysts for the production of fructooligosaccharides (FOSs). The crystal structure of EtLSC in complex with levanbiose (LBS) was obtained by soaking the preformed crystals in a solution containing a high concentration of sucrose followed by crystal storage in liquid N_2_. LBS is a di-fructose FOS intermediate formed during the synthesis of longer-chain FOSs. The binding pocket discovered in this crystal structure represents a starting point for planning mutagenesis studies in order to understand its biological relevance and role in FOS chain elongation [5]. 

Two major lipoglycans, lipomannan (LM) and lipoarabinomannan (LAM), are produced by *Mycobacteria*. Their structural characterization is very difficult due to their heterogeneity in terms of internal and terminal covalent modifications and branching patterns. To allow identification of the reducing end of these molecules, enzymes such as endo-α-(1→6)-D-mannanase from *Bacillus circulans* proved useful in cleaving the mannan backbone of LM and LAM. Angala et al. report the production and purification of the glycosyl hydrolase domain of the endo-α-(1→6)-D-mannanase gene from *B. circulans* TN-31 and studied its substrate specificity [6].

A GH3 β-glucosidase from the thermophilic fungus *Chaetomium thermophilum* was structurally characterized by Mohsin et al [7]. The structure provides useful information for determining a better use of enzymes in biotechnological applications for cellulose degradation in the production of biofuels, as thermophilic fungi are a promising source of enzymes with improved stability. The enzyme displays moderate glycosylation compared to other GH3 family β-glucosidases with similar structure, and a comparison of several thermostability parameters suggests that glycosylation and electrostatic interactions between charged residues may contribute to the enzyme’s stability at high temperatures. The structure provides insights into the GH3 enzyme family for further improvements of the β-glucosidases used in biotechnological cellulose degradation [7]. 

Two homologous uridine-5’-diphosphate (UDP)-glucose pyrophosphorylase enzymes produced by *Rhodococcus opacus*, 1CP—RoGalU1 and RoGalU2, their genes were cloned and the corresponding proteins characterized by Kumpf et al [8]. UDP-glucose is a versatile building block in both prokaryotes and eukaryotes. UDP-glucose is synthesized by the enzyme UDP-glucose pyrophosphorylase (GalU). The two enzymes are encoded by most *Rhodococcus* strains, known to synthesize natural products (i.e., trehalose-containing biosurfactants). Like other GalUs, RoGalU2 seems to be rather specific for the substrates UTP and glucose 1-phosphate, while dTTP and galactose 1-phoshate are substrates with merely 2% of residual activity. In comparison to other bacterial GalU, RoGalU2 activity was greater even at high temperatures [8]. 

The peptidoglycan of bacterial cell walls is hydrolyzed by muramidases/lysozymes, a lysozyme glycoside hydrolase (GH) family 22 C-type, from the upper gastrointestinal tract of the folivorous bird *Opisthocomus hoazin* in the native form and various mutants were expressed in *Aspergillus oryzae*. All mutants were enzymatically active; four of them with improved thermostability at pH 4.7, compared to the wild type. The X-ray structure of the enzyme was determined in the apo form and in complex, with chitin oligomers providing valuable information on substrate binding. Bioinformatic analysis of avian GH22 amino acid sequences showed that they split into three distinct subgroups, and the enzyme from *O. hoazin* is found in the “other birds” group. Taylor et al. propose that this represents a new cluster of avian upper-gut enzymes [9]. 

The use of deep eutectic solvents (DES) for the synthesis of alkyl glycosides catalyzed by the thermostable α-amylase from *Thermotoga maritima* Amy A was investigated by Miranda-Molina et al [10]. While DES containing alcohols, sugars, and amides as hydrogen-bond donors (HBD) as cosolvents performed best, pure DES almost completely deactivated the enzyme. Circular dichroism measurements of Amy A showed that large conformational changes were observed above 60 °C, changes not observed in aqueous medium. The authors claim that this is the first report on the effect of DES and temperature on an enzyme, to establish the temperature limit for a thermostable enzyme in DES [10]. 

The 15-O-glycosylation of ganoderic acid A (GAA) into GAA-15-O-β-glucoside is carried out by the enzyme BtGT_16345 from *Bacillus thuringiensis* GA A07. The enzyme was identified by whole genome sequencing of strain GA A07. A phylogenomic analysis revealed the species of the GA A07 strain to be *B. thuringiensis*. The enzyme is also regioselective toward triterpenoid substrates. BtGT_16345 showed glycosylation activity toward seven flavonoids (apigenin, quercetin, naringenin, resveratrol, genistein, daidzein, and 8-hydroxydaidzein) and two triterpenoids (GAA and antcin K) [11]. 

Roth et al. describe novel fungal amylases from *Thamnidium elegans* and *Cordyceps farinosa* with potential industrial applications, as they feature activity and high stability in a broad pH spectrum extending to pH 8 [12]. These enzymes have the typical GH13 α-amylase fold but feature shortened loops flanking the substrate-binding cleft, creating a large crevice. The inhibitor acarbose, in form of a transglycosylation product, was bound in the active site in the amylases from *T. elegans* and *C. farinosa*. Moreover, a potential novel binding site in the C-terminal domain of the *Cordyceps* enzyme was identified, which might be part of a starch interaction site [12]. 

A directed evolution approach was used by Yoav et al. to enhance the thermostability of *Clostridium thermocellum* β-Glucosidase [13]. In the process of cellulose utilization, β-glucosidases are key enzymes that convert cellobiose, produced by cellulose hydrolysis, to glucose. A thermostable mutant with kcat and *K*m, similar to those of the wild-type enzyme, was produced. The addition of the thermostable double mutant (A17S and K268N) to *C. thermocellum* secretome in order to carry out the hydrolysis of microcrystalline cellulose at 70 °C drastically increased the soluble glucose yield compared to the activity of the secretome supplemented with the wild-type enzyme [13]. 

A series of crystal structures of the wild type of the cold-adapted β-d-galactosidase from *Arthrobacter* sp. 32cB, as well as its mutant E441Q, in complex with various ligands were obtained in order to describe the reaction mechanism. Comparative analysis with mesophilic homologs revealed striking differences in the enzymatic reaction. Access of the substrate to the active site is facilitated by the fact that in ArthβDG a 10-aa loop is moved outward, while in mesophilic GH2 βDGs the same loop hampers access to the active site. The authors suggest that the exposure of the active site entrance improves the turnover rate of the cold-adapted enzyme [14]. 

Franceus et al. report the characterization of a sucrose 6F-phosphate phosphorylases member (SPP) of the family GH13 subfamily 18 from *Ilumatobacter coccineus* and its comparison with the enzyme from *Thermoanaerobacterium thermosaccharolyticum* [15]. Crystal structures of both SPPs were determined to provide insight into their similarities and differences. These enzymes are interesting because of their reversible phosphorolysis reactions in the synthesis of various glycosidic compounds. *I. coccineus* has stricter specificity than the promiscuous SPP from *T. thermosaccharolyticum*. Considerable differences between the two enzymes were found in the residues responsible for binding the fructose 6-phosphate group in subsite +1. Mutants that provided a higher degree of substrate promiscuity in the SPP from *I. coccineus* were probed, thus paving the way to rational enzyme engineering in biotechnology [15]. 

The degradation of pectins carried out by pectate lyases are of great interest in food and textile industrial production. An alkaline pectate lyase gene (*pppel9a*) from *Paenibacillus polymyxa* KF-1 belonging to the polysaccharide lyase family 9 (PL9) was cloned and the corresponding enzyme was expressed and characterized by Yuan et al [16]. The enzyme substrates are homogalacturonan-type (HG) pectins vis-à-vis rhamnogalacturonan-I (RG-I)-type pectins. The lyase activity was found to be optimal at pH 10.0 and 40 °C. The molecular mass of citrus pectin (~230.2 kDa) was reduced by PpPel9a action to ~24 kDa. PpPel9a was shown to be an endo-pectate lyase, acting primarily on the HG domain of citrus pectin. The enzyme was proposed to have potential in the preparation of pharmacologically active pectin products [16].

A xylanase belonging to family GH10 from the halo-thermophilic bacterium *Roseithermus sacchariphilus* strain RA (XynRA2) was characterized by Teo et al. [17]. XynRA2 is composed of a family 4_9 carbohydrate-binding module (CBM4_9), a family 10 glycoside hydrolase catalytic domain (GH10), and a C-terminal domain (CTD) for a type IX secretion system (T9SS). This study reports the identification and biochemical characterization of XynRA2 and its CBM-truncated variant (XynRA2ΔCBM). The mutant XynRA2ΔCBM showed a lower activity compared to the wild type, underlying the importance of XynRA2 carbohydrate-binding module in determining enzyme performance [17]. 

The review by Roman et al. reports an up-to-date analysis of the structural and functional diversity of β-xylosidases and discusses their inhibition by monosaccharides. β-xylosidases are crucial enzymes in biotechnology because they hydrolyze the glycosidic bonds of cellulosic biomasses with a high potential for degradation into reducing sugars, which can be used in the subsequent fermentation into bioethanol [18]. 

The review by Mestrom discusses the use of Leloir glycosyltransferases, as they offer excellent control over the reactivity and selectivity of glycosylation reactions. One-pot multi-enzyme glycosylation cascades have been achieved by the development of nucleotide-recycling cascades for the production and reuse of nucleotide sugar donors, and complex stereochemistry glycans and glycoconjugates can be constructed. Therefore, Leloir glycosyltransferases, on account of their reactivity and selectivity of glycosylation reactions with unprotected carbohydrates, are good candidates for biotechnological industrial applications in multi-enzyme glycosylation cascades [19]. 

In a review about the ketal-pyruvylation of monosaccharides, Hager et al. illustrate different classes of pyruvylated glycoconjugates and their associated functions, as well as the pyruvyltransferases responsible of the modification. Ketal-pyruvylation is present in diverse classes of glycoconjugates, produced in bacteria, algae, and yeast, but is absent in humans. The authors collected up-to-date information on the prevalent ketal-pyruvylation of monosaccharides, including specificity and sequence space of pyruvyltransferases, and provided insights into pyruvate analytics [20]. 
